# Obesity & Diabetes: An experience at a public sector tertiary care hospital

**DOI:** 10.12669/pjms.301.4314

**Published:** 2014

**Authors:** Zeeshan Ali, Syed Masroor Ahmed, Ayesha Nageen, Muhammad Tanveer Alam, Shabnam Sohrab

**Affiliations:** 1 Dr. Zeeshan Ali, Senior Registrar, Medical Unit III, Ward-7, Jinnah Postgraduate Medical Centre (JPMC), Karachi, Pakistan.; 2 Dr. Syed Masroor Ahmed, Associate Professor of Medicine, Medical Unit III, Ward-7, Jinnah Postgraduate Medical Centre (JPMC), Karachi, Pakistan.; 3 Dr. Ayesha Nageen, Postgraduate, Medical Unit III, Ward-7, Jinnah Postgraduate Medical Centre (JPMC), Karachi, Pakistan.; 4 Dr. Muhammad Tanveer Alam, Assistant Professor Medicine, DUHS & Civil Hospital, Karachi, Pakistan.; 5 Dr. Shabnam Sohrab, Senior Registrar Medicine, Medical Unit III, Ward-7, Jinnah Postgraduate Medical Centre (JPMC), Karachi, Pakistan.

**Keywords:** Diabetes, Obesity, Hypertension, Smoking

## Abstract

***Objective:*** To detect the frequency of Obesity in type 2 diabetic patients.

***Methods:*** It was a Cross Sectional study carried out at Diabetes Clinic, Medical Unit III, Jinnah Postgraduate Medical Centre Karachi from 1^st^ Jan 2012 to 30^th^ June 2012. Three hundred and eighty seven (387) type II diabetic patients of either sex and any age were included in the study. Non-purposive convenience sampling technique was used to enroll patients in the study. History regarding diabetes, hypertension (HTN), Cerebrovascular Accidents (CVA), smoking and other tobacco exposure was taken. Physical examination was carried out and height, weight, body mass index (BMI), blood pressure, peripheral pulses and ankle-brachial index (ABI) was calculated. Categorical variables such as Gender, Age groups, BMI groups, HTN, smoking, hyperlipidemia and ABI were expressed as frequencies and proportions. Means with standard deviations were calculated for continuous variables such as age, duration of diabetes, BMI, duration of HTN and duration dyslipidemia. For categorical variables, differences between patients were tested using the chi-square test. P value of ≤ 0.05 was considered significant.

***Results:*** Males were 128 in number (33%) and female were 259 in number (67%). Mean age was 52 yrs (+/- 9.67) and the mean duration of diabetes was 9.36 yrs (+/- 6.39). Hypertension was seen in 210 people (54%). 49(12.7%) were smokers and 39(10%) chewed tobacco. Normal BMI was seen in 62 patients (16%), 44 (11.4%) were overweight and 281(72.6%) was obese. Obesity was much more prevalent amongst the female gender that is 208(80%) versus male which was 73 (57%) and this was statistically significant (p-value 0.001). Hypertension was also more prevalent in obese patients and was statistically significant (p-value 0.04). Statistically significant lower mean BMI was found in smokers, tobacco chewers and/or had exposure to tobacco (0.001, 0.04, and 0.001 respectively).

***Conclusion:*** The study shows that there is a strong association of diabetes with obesity. Female gender had relatively higher BMI. Hypertension was more prevalent in obese diabetic subjects. Smoking and nicotine exposure was associated with significantly lower BMI.

## INTRODUCTION

Diabetes mellitus (DM) is a syndrome that is becoming prevalent globally at an alarming rate. It is increasingly presenting with complications of dire consequences like retinopathy, neuropathy, ischemic heart disease, nephropathy and diabetic foot ulcers. It is the most common non communicable global disease with 246 million people being inflicted with diabetes. According to International Diabetes Federation (IDF) it is the fourth leading cause of death.^1^

Pakistan is also bearing the burden of diabetes. Its prevalence is 6.74% as per IDF estimates in 2012 with an adult population having diabetes being 6550,000 (diagnosed) and 92000(undiagnosed), a total of 6.6 million.^2^

There is a strong association between abdominal obesity and diabetes. Obesity is now understood as a leading cause of diabetes. Obesity among the world population is also becoming a pandemic. Greater than 2.5 million deaths are now related to obesity. Its prevalence has raised three fold since 1980. According to the WHO it is now a global epidemic both in the developed and underdeveloped nations. About 320 million are obese in the world with a BMI of greater than 30 and 1.1 billion greater than BMI of 25.^3^ Obesity is increasing mainly due to the growing trend of a sedentary lifestyle and high caloric diet.

The increase in diabetes is closely linked to upsurge in obesity. Compared to lean persons obese men and women of BMI greater than 35 have a 60 and 90 fold increases in the possibility of developing diabetes, respectively.^4^ Obesity exacerbates the insulin resistance of the body aggravating diabetes. Studies suggest that the Body Mass Index, waist/hip ratio and waist circumference are linked and associated to the development of diabetes eventually.

The effect of obesity in diabetic patients is that it increases their morbidity and mortality. The concomitant increase in both diabetes and obesity has lead to an increase in incidence of coronary heart disease which could be due to a high lipid profile. About 80% of diabetic obese people will die prematurely of cardiovascular diseases.^5^

Obesity is preventable and reversible if there is awareness to the problem and its consequences. If steps are taken early and appropriately weight loss can be treated and maintained.

It is a challenge for an obese patient to achieve a sustainable weight loss and yet maintain a normal regular daily routine. This can be achieved by calculated correct diet and lifestyle advice and support. Expert opinion has to be given regarding exercises. It is seen that outcome is more effective if a better understanding of energy metabolism is given. Frank discussion with patients is needed to solve individual issues regarding diet and lifestyle alteration. Realistic target goals are to be set. If needed there are synthetic food diet available of 400kcal/day.^5^ Our objective was to document frequency of obesity in patients with Type-2 diabetes who were visiting this public sector tertiary care facility.

## METHODS

This cross sectional study was conducted at the Diabetes Clinic of Medical Unit III, Jinnah Postgraduate Medical Centre Karachi. A total 387 adult patients of diabetes mellitus (DM) type II were enrolled using non-purposive convenience sampling technique. Patients of either gender, any age or duration of DM were included in the study. 

After obtaining an informed consent, a detailed history regarding diabetes, hypertension (systolic B.P> 140 mmHg and diastolic B.P> 90 mmHg on examination or diagnosis of HTN taking antihypertensives), CVA (any focal neurological defect on examination or previous history or admission of radiologically proven diagnosis of stroke), smoking and other tobacco exposure was taken. Physical examination was carried out by a research associate and basic anthropometric data; height, weight, body mass index (BMI), blood pressure, peripheral pulses and ankle-brachial index (ABI) was calculated. On the basis of WHO recommended Asian cut off values for BMI kg/m^2^; patients were divided in to three groups; Normal (18.5 to 22.99), overweight (23 to 24.99), and obese (≥25). The patients were also grouped according to age and duration of diabetes. The ABI was calculated and classified according to the criteria as mentioned by American Diabetes Association (ADA).^6^

Data was collected and recorded on a pre-designed proforma and analyzed by SPSS v.19. Categorical variables such as Gender, Age groups, BMI groups, HTN, smoking, hyperlipidemia and ABI were expressed as frequencies and proportions. Means with standard deviations were calculated for continuous variables such as age, duration of diabetes, BMI, duration of HTN and duration dyslipidemia. For categorical variables, differences between patients were tested using the chi-square test. P value of ≤ 0.05 was considered significant.

## RESULTS

A total of 387 patients were enrolled. All were type 2 Diabetes. Males were 128 in number (33%) and female were 259 in number (67%). Mean age was 52 yrs (+/- 9.67) and the mean duration of diabetes was 9.36 yrs (+/- 6.39).

History of Ischemic heart disease was present in 70 patients (18%) and an episode of stroke had occurred in 9 patients (2.3%). Dyslipidemia was there in 105 patients (27%). Hypertension was seen in 210 people (54%). 49(12.7%) were smokers and 39(10%) chewed tobacco.

Mean Body mass Index was 28.02 (+/- 5.31). Obesity was classified according to Asian criteria into three groups. Normal (BMI <22.9), overweight (BMI<23-24.99) and obese (BMI>25). Normal BMI was seen in 62 patients (16%), 44 (11.4%) were overweight and 281(72.6%) was obese.

Fifty nine patients (15%) were on insulin treatment only while the rest were treated with oral hypoglycemic. 44 out of 59 patients (74%) patients taking insulin were obese while 237(72%) of patients taking oral hypoglycemic were obese. No significant difference in mean BMI was seen regarding the mode of treatment either by insulin and oral hypoglycemic. Amongst the patients on oral hypoglycemic majority of patients were on dual therapy (metformin and glibenclamide). Patients taking insulin only 11(2.8%) were exclusively on insulin and the rest were on triple therapy. Mean BMI of those patients who were on only insulin therapy was slightly lower 26.99 versus those on oral hypoglycemic 28.05 but it was not statistically significant. There was no difference between the mean BMI of age groups and p value was not significant (0.2).

Obesity was much more prevalent amongst the female gender that is 208(80%) versus male which was 73 (57%) and this was statistically significant (p value 0.001). Hypertension was also more prevalent in obese patients that is 162 out of 210 (77%) hypertensive were obese while 119 out of 177 (67%) of non hypertensive were obese. This observation also had statistically significant value (0.04). Peripheral artery disease was equally prevalent amongst obese and nonobese patients, although slightly more in obese patients but not statistically significant.

The exposure to tobacco either as smoking cigarette or as chewing tobacco was found protective as statistically significant number of patients smoking were having lower mean BMI than who were not smoking. The same was true for chewing tobacco. Amongst the smoker 23 out of 44 (46%) were obese while it was found that those not smoking 258 out of 338 (76%) were obese. Similarly those who were chewing tobacco 22 out of 39 (56%) were obese while those who were not chewing tobacco 259 out of 348 (70%) were found to be obese. When those two factor were combined the total 81 patients had total tobacco exposure out of which 41 out of 50 (20% of the total study population) were obese while in the group not taking tobacco exposure, it was 240 out of 306 (78%). P value for smoking, chewing tobacco and tobacco exposure were statistically significant that is 0.001, 0.04, and 0.001 respectively.

## DISCUSSION

There is a strong association between abdominal obesity and the development of type 2 diabetes.^7^ The study was done on 387 diabetic patients analyzing the association between diabetes and obesity. The age distribution of the patients showed that 164(42.4%) were less than 50 yrs, 137 (35.4%) were between 50-60 yrs and 86(22.2%) were greater than 60 years.

**Table-1:** Body Mass Index Compared in Different Risk Groups

		*BMI Groups*						
		*Normal*		*Over Weight*		*Obese*		*p-value*
Age Groups	< 50 years	24	15%	17	10%	123	75%	0.664
	>= 50 years	38	17%	27	12%	158	71%	
Gender	Male	32	25%	23	18%	73	57%	0.001
	Female	30	12%	21	8%	208	80%	
Hypertension	yes	25	12%	23	11%	162	77%	0.044
	no	37	21%	21	12%	119	67%	
Duration of Diabetes Groups	<= 5 years	24	19%	16	12%	88	69%	0.473
	> 5 years	38	15%	28	11%	193	75%	
Duration of Hypertension Groups	<= 5 years	10	10%	15	14%	80	76%	0.206
	> 5 years	15	14%	8	8%	82	78%	
Smoking	yes	16	33%	10	20%	23	47%	0.001

**Table-II:** Descriptive Statistics

	*N*	*Minimum*	*Maximum*	*Mean*	*Std. Deviation*
Age (years)	387	22	76	52.22	9.671
Duration of Diabetes	386	.5	35.0	9.387	6.3998
CAD Duration	59	.3	20.0	6.376	5.1126
Stroke Duration	9	1.0	6.0	2.333	1.5000
Dyslipidemia Duration	56	.2	15.0	4.373	3.7230
Hypertension Duration	197	.2	27.0	7.856	6.4156
Smoking Duration	46	1.0	50.0	20.783	14.2094
BMI	387	17.36	55.43	28.0226	5.31882

Males in the study were 128 (33.1%) and females were 259(66.9%) showing that females presented more with diabetes. With respect to the main objective of the study the results were that diabetic patients with normal weight were 62 (16%), overweight were 44 (11.4%) and obese were 281(72.6%). Thus, together the overweight and obese were 325 people (84%) confirming the association of diabetes with obesity.

In a study done in Liverpool, United Kingdom 86% of diabetic patients were overweight which was similar to our results.^8^ In our study when Asian cutoffs were applied, underweight were 3 (0.8%), normal) were 59 (15.2%), overweight were 44(11.4%), obese class 1 were 163(42.1%), obese class 2 were 118 (30.5%) pointing the fact that obesity was more prevalent in diabetics.

Looking in to the gender difference in diabetics regarding weight, in the males, 32 out of 62 (51.6%) were of normal weight and in females they were 30 in 62(52.3%). Overweight males were 23 in 44 (52.3%) overweight females were 21 in 44 (47.7%) and obese males were 73 out of 281(26%) while obese females were 208 out of 281(74%). This showed that females were significantly more in the obese group.

A local study done in Peshawar in 2006 on the obesity profile of Khyber Pakhtunkhwa also showed that 67% of the obese were females with 68% being of urban area emphasizing the role of sedentary lifestyle and dietary habits in urban area.^3^ In a local study in AKUH, Karachi conducted in 1990 of the 25% overweight people one half were women and one third were men again showing females to have a greater tendency to be obese and hence diabetes.^9^

Obesity is a strong independent factor for hypertension. High blood pressure is also associated with an increase in body weight. In Framingham study prevalence of hypertension among obese was twice that of normal weight people irrespective of age and gender. Similarly in our study, hypertension was found in 25 out of 62 (40.3%) of normal weight diabetics and 23 out of 44 (52.3%) of overweight 162 out of 281 (57.7%) of obese patients.

Analyzing the age and obesity, in the <50 age group, 24 out of 62 (38.7%) were of normal weight, 17 out of 44 (38.6%) were overweight and 123 out of 281(43.8%) were obese denying any change in trend in obesity within the younger group. In the 50-60 age group patients of normal weight were 18 out of 62 (29%) and overweight were 18 out of 44 (40.9%) and obese were 101 out of 281 (35.9%) displaying more overweight people in the 50-60 years. In patients older than 60years, there was not much difference in the results with normal weight patients being 20 in 62 (32.3%), overweight being 9 in 44 (20.5%) and obese being 57 out of 281(20.3%). Thus, there is a tendency to be overweight in the 50-60 years age group.

In a local study conducted in Peshawar, the age group which showed obesity was younger around 35-45 years.^10^ However, a national survey of Pakistan showed most obesity in the age group of 45-64 years with 40% being females and 23% males.^9^

The diabetic patients were also grouped according to the duration of diabetes to see if there is any association of diabetes duration with obesity. In the category of patients with less than five years of diabetes, normal weight were 20 out of 62 (32%), overweight were 14 out of 44 (31%) and obese were 70 out of 281(25%). In the five to nine years old duration the patients with normal weight were 14 out of 62 (22%), overweight were 12 out of 44 (27%) and obese were 104 out of 281(37%). In the greater to nine years group the diabetics with normal weight were 28 out of 62(45%), overweight were 18 out of 44(41%) and obese were 107 out of 281 (37%). Thus there was no special finding regarding the duration of diabetes with weight in the diabetics. 

Obesity and diabetes are components of metabolic syndrome.^11^ Obesity increases the risk of cardiovascular disease in adults and has been strongly associated with insulin resistance in normoglycemic persons and in individuals with type 2 diabetes.^11^^,^^12^

 In our study we found an increase in the frequency of history of ischemic heart diseases in obese people. In normal weight people the percentage was 8 out of 62 (13%), in overweight 6 out of 44 (13.6%) and in obese in 56 out of 281 (19.9%). These people had history of IHD either angina or Myocardial infarction.

Similarly, the frequency of peripheral artery disease in normal weighted patients was 56 out of 281(33.9%), overweight in 16 out of 44 (36.4%) and obese in 115 out of 281 (40.9%) showing the atherosclerotic complications in diabetes with obesity. There finding were though not statistically significant but showed the trend of increasing complication with increasing BMI.

Another important observation was of dyslipidemia. In diabetic patients there is 13% increase in serum cholesterol level compared to control. The adipose tissue related proteins are highly expressed in diabetic obese compared to normal.

The important protein leptin interferes with glucose homeostasis which is present in diabetic obese people. The most common component of metabolic syndrome is low HDL or high LDL.^1^ The results showed 16 out of 62 (25.8%) being of normal weight, 7 out of 44 (15.9%) being overweight and 82 out of 281(29.2%) obese subjects had dyslipidemia.

Another observation was that patients that smoked had a lower mean BMI (46% were obese) compared to nonsmokers (76% were obese). This is in aggreement with studies worldwide where there is a growing awareness about the beneficial effect of smoking on weight.

There was male predominance in the smoker group 39 out of 49 (80%) tobacco exposure 53 out of 81 (65%) and in our study male gender had statistically significant lower BMI but this was not true for tobacco chewing as it was equally present in both groups 19 out of 39 male and 20 out of 39 were female. 

Nicotine is an appetite suppressant. The act of smoking triggers behavior modification that prompts smokers not to have snacks.^13^ similarly chewing tobacco also had the same effect as there were 56% obese in the group that chewed and 70% obese in the group that didn’t. There is a growing epidemic of obesity and type 2 diabetes in the world, more than 75% of the patients are in the developing countries.^14^^-^^17^

Overweight and obesity were significantly associated with diabetes, hypertension, high cholesterol, asthma, arthritis, and poor health status.^18^^,^^19^ The scales are certainly tipped in favor of losing weight and living a healthy lifestyle. This can be achieved by a calculated correct diet, lifestyle advice and support, exercise program, patient education and time set target goals. It would be then that many serious diseases can be prevented or at least delayed by moving towards a more normal weight.^20^

## CONCLUSION

The study shows that there is a strong association of diabetes with and obesity. Female gender had relatively higher BMI. Hypertension was more prevalent in obese diabetic subjects. Smoking and nicotine exposure was associated with significantly lower BMI. 

## Author Contributions:

**ZA** conceived the idea and did statistical analysis.

**SMA** Critically reviewed the manuscript and corrected it for publication.

**AN** contributed in manuscript writing and data collection.

**SS** Tabulated the results and contributed in statistical analysis.

**MTA** did the literature search and contributed in manuscript writing.
